# Impact of Protein Concentrate Production on Mycotoxin Mitigation: A Systematic Review

**DOI:** 10.3390/toxins17120572

**Published:** 2025-11-26

**Authors:** Caroline Senna, Marianna Cruz, Larine Kupski, Eliana Badiale-Furlong

**Affiliations:** School of Chemistry and Food, Federal University of Rio Grande, Av. Italy km 8, Carreiros, Rio Grande 96203-900, Brazil; carolalmeidasenna@gmail.com (C.S.); dqmebf@furg.br (E.B.-F.)

**Keywords:** aflatoxins, Ochratoxin A, mitigation, proteins, zearalenone

## Abstract

The plant-based protein industry has explored new material sources, such as agro-industrial by-products and extraction techniques based on chemical properties assisted by ultrasound, high pressure and other tools to improve the yield and functionality of protein concentrates. However, promising by-products from vegetable processing are susceptible to incidental and natural contaminants, mainly mycotoxins. Adopting sustainable strategies and understanding how they affect mycotoxin fate during processing remains a challenge to ensure food security. In this study, a systematic literature review and bibliometric analysis were conducted to identify reliable pre-treatments and treatments for producing protein concentrates and evaluate the efficiency of technologies to mitigate mycotoxin bioaccessibility. Searching for research in Scopus, Web of Science and ScienceDirect (2010–2024) identified 3688 scientific articles on techniques to improve the yield and functionality of recovered proteins, but only three studies addressed mycotoxin fate. Aflatoxin, the most prevalent mycotoxin in raw materials, was the only one considered, highlighting that chemical and enzymatic treatments may help mitigate mycotoxicological risks in protein concentrates. Results indicate a gap in plant-based food security regarding mycotoxin contamination, which must be addressed through mitigation strategies aligned with efficient processes to ensure sustainable and safe plant protein-based foods.

## 1. Introduction

The growth of the global population and changes in food habits have led to a search for quality and quantity in food patterns, such as trends towards plant-based protein in food consumption [1], despite their nutritional limitations regarding the balance and digestibility of amino acids [2]. The vegetable protein industry has grown and requires new sources of protein beyond traditional legumes (soybeans, peas, chickpeas, and beans), cereals (rice, wheat, corn, and oats) and oilseeds (sunflower, flaxseed, sesame, and peanuts) [3].

To meet this demand, the sustainability of food supply chains must be improved using efficient methods to extract proteins from conventional and new sources. Proteins have been extracted using techniques such as extraction with organic solvents, alkaline extraction [4,5,6], enzyme-assisted extraction [4,7], ultrasound-assisted extraction [8], microwave-assisted extraction [9], extrusion [4], high-pressure extraction [10], drying and combinations of enzymatic hydrolysis and extrusion [4]. These techniques have advantages and disadvantages; however, their main targets are the performance and functional properties of proteins [4].

However, the dietary trend based on plant protein may represent a new gap in food safety due to grain susceptibility to fungal contamination, a fact that has concerned re-searchers [11]. The Food and Agriculture Organization (FAO) has stated that approximately 25% of grains produced worldwide are contaminated with different mycotoxins, isolated and conjugated, because of fungal contamination in different stages of the food supply chain [12,13,14,15].

Mycotoxins are toxic secondary metabolites produced by various filamentous fungi. They have low molecular weights and persist not only in food production chains, but also in processed food [16,17]. Studies on the occurrence of mycotoxins in food have emphasized the detection and quantification of aflatoxins (AFs), fumonisins (FBs), zearalenone (ZEN), trichothecenes (TCT) and patulin (PAT), which cause adverse effects such as imunotoxicity, carcinogenicity, nephrotoxicity, hepatotoxicity, neurotoxicity and teratogenicity, and may also damage the environment and socioeconomic systems [18]. Owing to their chemical and thermal stability, it is difficult to decontaminate food throughout its processing stages [19,20], which has led to studies on decontamination strategies.

Conventional strategies for decontamination include physical, chemical, and biological treatments to promote mycotoxin degradation or removal and mitigate harm caused by dietary exposure [21,22,23]. Despite the variety of extraction and pretreatment techniques used for producing protein concentrates, such as ultrasound, enzymatic hydrolysis and pH treatments, their potential roles in mitigating mycotoxin contamination has not been thoroughly explored. Whether these treatments may lead to reductions in detectable mycotoxin levels or bioaccessibility within protein concentrates and isolates remains unclear. These questions guided the objectives of this systematic literature review and bibliometric analysis, which aimed to identify reliable pre-treatments and treatments used for producing protein concentrates, investigate the main raw material employed to produce protein concentrates, and evaluate the reduction in mycotoxin levels achieved by different treatments.

## 2. Results

### Systematic Review Process and Bibliometric Analysis

**Figure 1:**
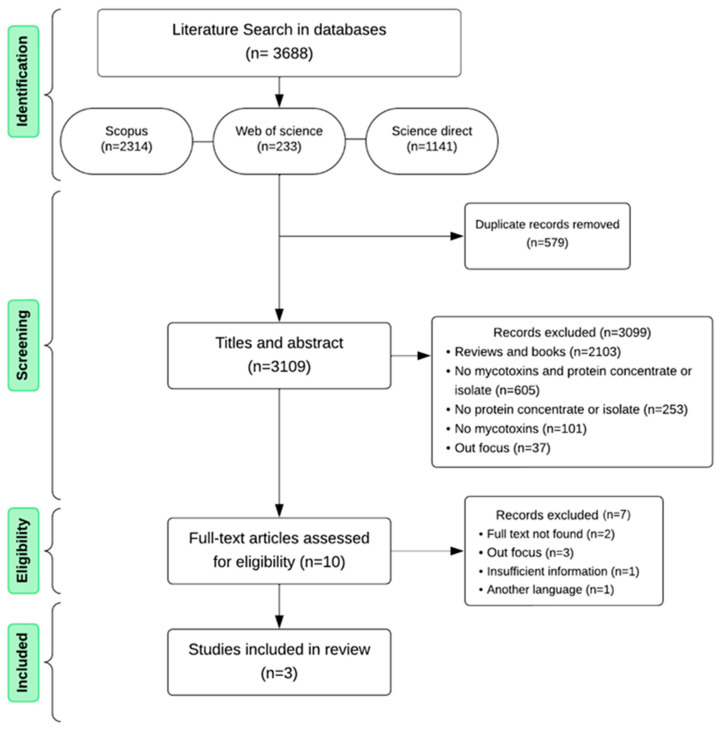
Diagram of the systematic review.

The systematic search led to 3688 results (Figure 1): 2314 in Scopus, 233 in Web of Science, and 1141 in Science Direct. Duplicate results were checked and 579 papers were removed. In the screening stage, titles and abstracts were evaluated and 3099 records were excluded: 2103 were reviews and books, 605 were not specific to mycotoxin and protein concentrates and isolates, 253 focused on neither concentrates nor isolates, 101 were not about mycotoxins and 37 were not within the scope of the review. The excluded studies in the eligibility stage were those that dealt with: (a) animal supplementation with contaminated protein concentrates to evaluate DNA changes [24]; (b) oxidative stress induced by mycotoxins in cells [25]; (c) validation of the analytical method to quantify mycotoxins in protein concentrates [26,27]; and (d) other topics outside the review scope.

After screening, ten full-text articles were assessed for eligibility and seven records were excluded: two full texts were not found, three were out of the scope since they were about analytical method validation, one had insufficient information about analyzing mycotoxins in the raw material to obtain the concentrate (making it impossible to calculate the reduction) and one was not in English language. The quantitative results of search showed that there are relatively few studies that answer the question of this review. This is a reason for concern, considering the trends in vegan diets and plant-based protein markets.

**Figure 2:**
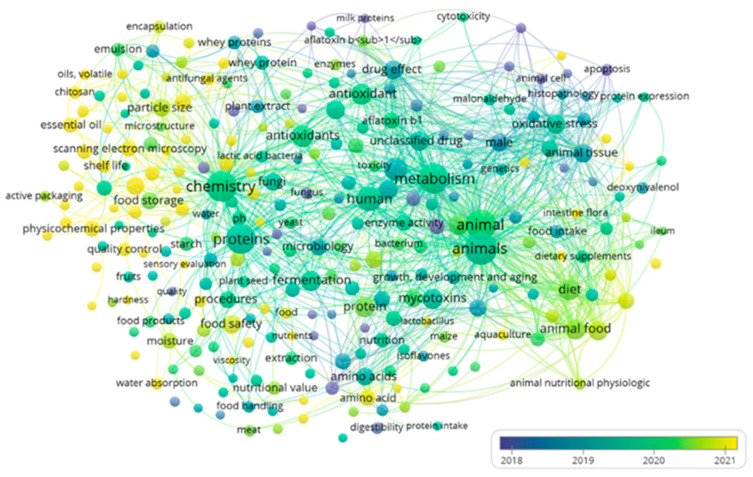
Word cloud of selected papers in the “screening” stage.

**Figure 3:**
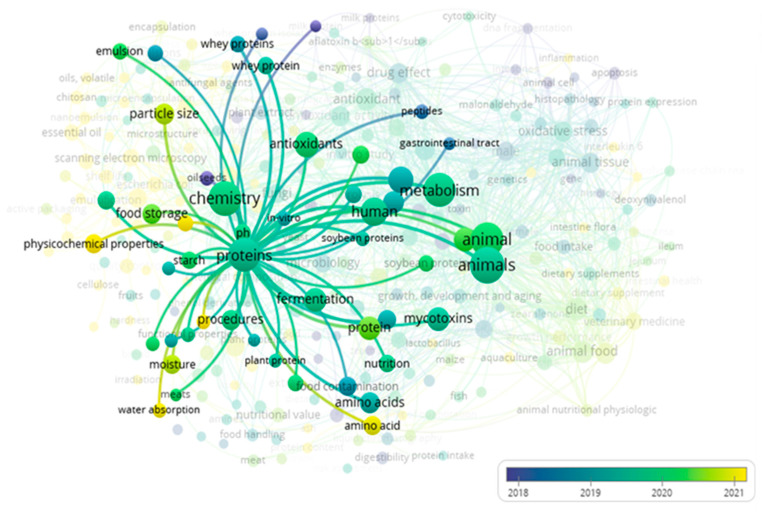
Interaction between keywords and “proteins”.

Subsequently, to construct the word cloud, papers in the screening stage (titles and abstracts) were used to visualize their main keywords after the combinations were applied. The word cloud highlights the importance of using the PICO strategy to develop a systematic review and visualize interactions among relevant terms of interest. The main keywords in Figure 2 were “chemistry,” “proteins,” “mycotoxins,” “metabolism,” and “animal.” When the keyword protein is highlighted (Figure 3), the main keywords that interact with “proteins” are: “mycotoxins,” “antioxidants,” “human,” “metabolism,” “chemistry,” “whey proteins” and “procedure” procedure’. This analysis demonstrates that existing studies relate mycotoxins to proteins, metabolism, and processes—confirming that this systematic review can address the guiding research question.

## 3. Discussion

Protein sources used to satisfy human metabolic needs are of animal origin, such as milk and meat, or plant-based sources such as seeds, beans, grains and nuts [28]. In addition, there are fungi, algae and insects [29]. Changes in consumption patterns, such as the rise in vegan and vegetarian diets, have led to the development of sustainable and friendly techniques for producing protein concentrates and isolates from traditional and alternative sources. Development of plant-based proteins has also been associated with long-term economic and environmental benefits, as some agro-industrial by-products are rich in proteins and may be better utilized [30].

Protein-extraction strategies using chemical [9,31], biochemical [32], physical [9,31,33,34] and physicochemical assisted-solubilization treatments have been applied individually or in combination [9,35] to enhance the yield, nutritional quality and technological properties of protein concentrates. Although these methods show promising results under experimental conditions, their implementation in large-scale production systems remains challenging [36].

Among the treatments used to extract proteins from different raw materials, the combination of proteolytic enzymes and other physicochemical processes has gained interest because of the hydrolytic cleavage of peptide bonds, which increases the free amino acid profile and carboxyl groups to enhance solubility [37], digestibility [38], and functional properties [39] through the generation of bioactive peptides [34]. Hydrolysis of lignocellulosic material by cellulolytic enzymes can increase the protein yield and improve the nutritional and technological properties of protein concentrates and isolates by breaking the association between proteins and lignocellulose in plant matrices [4,40].

It should be highlighted that raw materials used for protein extraction, such as grains and cereals, are susceptible to fungal contamination throughout their production chains [16,17]. This must be considered before incorporating these sources into plant-based dietary recommendations. Fungi not only degrade original matrices, but also include toxigenic species that produce mycotoxins under various biotic and abiotic conditions. They are stable structures, even under parameters capable of altering other molecular constituents of food matrices and their degradation products may be highly toxic [23,41].

Mycotoxin-producing fungal genera, such as *Fusarium*, *Aspergillus*, *Penicillium*, and *Alternaria*, are of global concern because they may contaminate agricultural raw and processed products from the field, production, distribution and consumption [42,43,44]. A study of mycotoxin contamination in cereal-based feed and food carried out by Palumbo et al. [42] showed that the highest concentrations in food were those of FBs, deoxynivalenol (DON), AFs, and ZEN found in wheat, corn and other grains used for producing protein concentrates. The occurrence was reported to be 54.9% of the total raw material, which means that they were co-contaminated with at least two mycotoxins.

**Figure 4:**
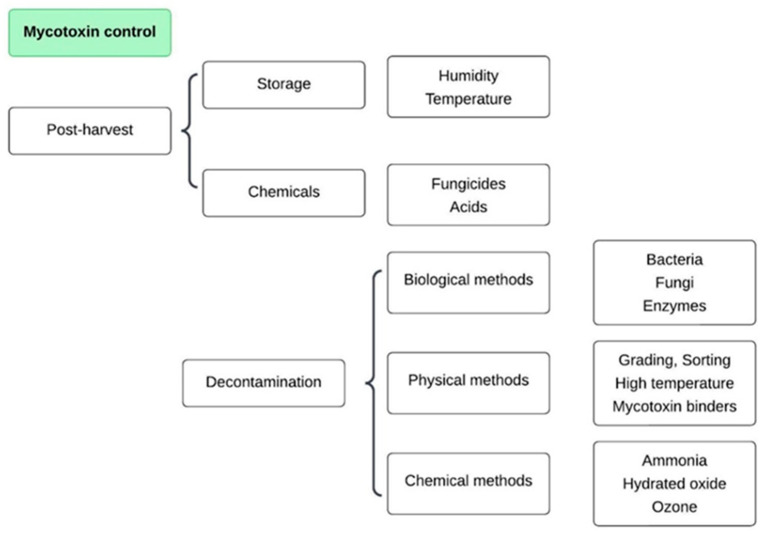
Methods of mycotoxin decontamination.

Consumption of food contaminated with mycotoxins can pose acute and chronic health risks, including hepatic disorders, immunosuppression and impaired growth. In the context of protein concentrates, the key safety implication is ensuring that these ingredients meet regulatory limits. Their incorporation into food formulations may contribute to overall dietary exposure. Thus, consistent monitoring is essential to guarantee the safety of the final products [45,46,47,48,49]. In light of this risk, various chemical, physical and biological decontamination techniques have been applied to the pre- and post-harvest stages, storage and processing. Piotrowska [23] discussed the main strategies for mycotoxin decontamination that are employed throughout food production chains (Figure 4).

Screening for fungal damage in grains is the starting point for applying the most traditional physical methods for decontaminating raw materials, including thermal treatments [50], UV radiation [51], cold plasma [52], electron beam irradiation [53], pulsed electric fields [54], and adsorbents [55,56]. Chemical methods are based on the addition of oxidants such as hydrogen peroxide, sulfur dioxide, sodium hypochlorite, ozone, and ammonia, which degrade the structures of mycotoxins [57,58,59]. These methods require further study because their results are controversial and depend on the type of mycotoxin and the material to which they are applied. In addition, they may generate toxic residues and affect the nutritional value and functional properties of products [41,58].

Techniques used for producing protein concentrates involve principles similar to those applied to the process of mycotoxin decontamination. These include chemical, physical, or biological treatments that modify the structure, solubility, or interactions of molecules. For example, pH shifts and solvent systems alter protein–matrix interactions in a manner comparable to how they disrupt mycotoxin binding; physical processes such as heating or phase separation can reduce the stability or partitioning of undesirable compounds; and enzymatic or microbial actions can selectively degrade or transform specific components [41,60]. It should be pointed out that, to understand how mycotoxins are associated with or bind to macromolecules in protein sources, it is fundamental to establish protocols to produce protein concentrates under parameters that reduce the bioaccessibility of mycotoxins originally found in the sources. Table 1 shows the selected studies that evaluated the occurrence of mycotoxins in protein concentrates and determined the reduction in the original contamination levels.

Several studies have emphasized the occurrence of AFs in peanuts used to produce protein products. Oakes et al. [55] evaluated the effect of spray drying on the production of protein hydrolysates from peanut flour (PF) contaminated with AFs (B1 + B2 + G1 + G2) and achieved a reduction in AFs from 41 to 99%. Various experiments have been conducted as pre-treatments to yield hydrolyzed PF, including acid and basic treatments (pH), the use of adsorbents (clay), and enzymatic hydrolysis (pepsin and Alcalase). Spray drying was used to produce dried protein hydrolysates.

Treatments used by Oakes et al. [55] showed that when they used pH variation and acidic (2.1) and basic (9.1) conditions, the resulting concentrations were 34.8 and 20.1 µg kg^−1^, which represented reductions in AFs of 41% and 65.9%, respectively. The inclusion of an adsorbent (clay) in the pretreatments resulted in a reduction of 87.8%. In the treatment that uses pH 9.1 combined with clay, AF was not detected (LODm < 1ppb). Spray drying reduced the AF levels to below those recommended by the Food and Drug Administration (FDA) (20 μg kg^−1^) in all treatments involving the addition of clay. Notably, treatments involving the use of Alcalase and clay resulted in levels below 1 μg kg^−1^ [55]. The combination of chemical treatments and spray drying further enhanced the AF reduction by up to 91.3% of the original contamination.

Studies of enzymatic hydrolysis to produce protein concentrates have been carried out and its effects on the detoxification of mycotoxins have been investigated [55]. The adsorption capacity of clay was demonstrated in an in vitro and in vivo study conducted by Kabak et al. [62]. Bentonite clays, such as AB20A, bind strongly to AFs through interactions with polar functional groups, such as AF [63].

A study conducted by White et al. [56] aimed to develop a pilot-scale process to produce PF hydrolysates with reduced AF levels and enhanced bioactive properties, starting from PF contaminated with AF (B1 + B2 + G1 + G2) at the concentration of 191 μg kg^−1^. First, an enzymatic pretreatment was applied to the PF samples using Alcalase hydrolysis. The liquid containing the hydrolysate was used to produce protein and to evaluate the AF content (B1 + B2 + G1 + G2).

Subsequently, other treatments that combine enzymatic hydrolysis, adsorption (clay), and centrifugation were applied. The first and second treatments involved varying the re-addition of Novasil from 4% to 8%, achieving reduction average of 98% (2.7 μg kg^−1^) in AF by comparison with the AF level in the raw material (191 μg kg^−1^ of AF). The results showed that an increase in the amount of Novasil did not significantly affect the reduction. The third and fourth treatments varied the percentages of AB20 clay re-addition from 4% to 8%, showing reductions in AFs of 92.8% and 94%, respectively, which were the highest among the treatments under investigation, indicating that re-addition of 8% AB20 resulted in the highest AF reduction.

Treatments to which clay was added showed a significant reduction in AF through adsorption, in accordance with the studies conducted by Kannewischer et al. [64] and Oakes et al. [55]. The authors also demonstrated that AF adsorbed on clay surfaces may be more susceptible to degradation than the free AF. The use of centrifugation also promoted the clarification of liquid hydrolysates, resulting in a reduction in AF concentrations to levels below those recommended by the FDA. Thus, centrifugation combined with adsorption has been confirmed to be a promising strategy for reducing AF in protein concentrates, although little has been evaluated regarding their nutritional value.

Zein, a group of proteins derived from the endosperm of corn kernels, can be sourced from three main types of corn materials: dry-milled corn (DMC), dried distillers grains with solubles (DDGS ) and corn gluten meal (CGM), which possesses a high protein content ranging from 62% to 74%. It should be highlighted that corn is prone to contamination by mycotoxins, such as ZEN, in its free form or associated with zein [61,63]. Tan et al. [61] evaluated the effects of different pH conditions (0, 1, 2, 7, 11, 12, 13 and 14) and temperatures (40 °C and 80 °C) on the functional properties of zein and levels of total ZEN, free ZEN and zein-bound ZEN. In addition to evaluating the effects on mycotoxin levels, the functional properties of zein, such as emulsifying activity, foaming capacity and stability, were also evaluated. This is interesting because decontamination methods may affect these properties and their applications, becoming a problem in the process.

The authors used an ethanol solution (80:20) for zein extraction and the pH of the mixture was adjusted by adding hydrochloric acid (HCl) and sodium hydroxide (NaOH). Subsequently, the different zein samples were extracted by continuous stirring (500 rpm) for 5 h at 40 °C and 80 °C. Proteins were precipitated by the addition of water, followed by washing with ultrapure water (three times) and centrifugation. After the extraction, ZEN was quantified. Increasing the pH from 7 to 11, 12, 13 and 14 promotes the opening of the lactone ring of ZEN, which is unstable in an alkaline environment [65]. Consequently, there was a significant reduction in the total ZEN detected. Even though pH 14 is optimal for mycotoxin degradation, it is not ideal for food formulations. Therefore, the effect of the temperature was evaluated. There was a greater reduction of 12.7% in total ZEN at 40 °C and 63.6% at 80 °C. Zein extraction at pH 14 and 80 °C may be an efficient method of decontamination to produce proteins with low levels of total ZEN. Another important factor observed in this study is that the emulsifying activity of zein extracted at pH 14 and 80 °C was higher than that of zein extracted at 40 °C. The foaming capacity and foam stability were not significantly affected by the protective conditions of temperature and pH, demonstrating that in addition to the reduction capacity of ZEN, it does not affect the functional properties of the extracted proteins.

However, there is a shortage of studies on the interactions between proteins and mycotoxins, such as ZEN, as well as the nutritional and technological effects of such treatment on the resulting zein. It should be emphasized that a high pH for food products is not recommended because it decreases the nutritional value through the degradation of proteins and vitamins [4].

Experimental data suggest that the types of pre-treatments and treatments used in protein extraction have the potential to reduce the detected mycotoxins, but they require further investigation into their safety, bioaccessibility and applicability for food use, despite demonstrating a reduction in AF and ZEA levels. Moreover, the results in Table 1 show that the combined strategies for chemical, enzymatic, and adsorption treatments proposed by Oakes et al. [55] and White et al. [56] (Table 1) promoted reduction in AF and ZEN, respectively, suggesting that enzymatic hydrolysis is beneficial when combined with other treatments during protein extraction from the raw material used in the process. Nevertheless, there are gaps in the literature regarding interactions between proteins and mycotoxins, their binding mechanisms, bioaccessibility and technological and nutritional consequences in the resulting concentrates and isolates. Notably, that chromatographic methods are the primary strategies for mycotoxin detection due to their high sensitivity; however, sample preparation represents a critical step affecting analyte recovery, and the interaction of mycotoxins with protein-rich matrices requires further investigation to accurately determine their distribution during protein concentrate production.

Bioaccessibility provides information on the actual health risks associated with exposure to contaminants, such as mycotoxins, since it enables the estimation of the proportion of ingested contaminants that may be released in the gastrointestinal tract and thus become available for absorption [66]. Therefore, knowledge of bioaccessibility is a key factor in assessing processes aimed at reducing and/or decontaminating mycotoxins in food, mainly in relation to those that represent a new nutritional trend. To propose mitigation strategies, they must be evaluated because mycotoxins may interact with the protein matrix or exist in masked forms, which affects the accurate determination of their levels in food using conventional analytical methods [41].

The reduction in toxicity may be inferred by a decrease in the bioaccessibility of mycotoxins, rather than solely relying on a reduction in their detectable levels in food [22,67]. Therefore, it is important to highlight an existing gap in the studies that were reviewed, indicating that bioaccessibility assessment would be beneficial to confirm the reduction in mycotoxin levels in protein concentrates produced by the authors.

Mitigation of mycotoxins in protein products can be achieved by integrating chemical, enzymatic, and adsorption-based treatments. Adjusting pH, using enzymes such as Alcalase and incorporating clay adsorbents effectively reduce aflatoxin and ZEN levels, particularly combined with processes like spray drying or centrifugation. These combined strategies not only decrease detectable toxin levels, but may also influence their bioaccessibility and interactions with the protein matrix, which are critical for assessing actual health risks. For industrial applications, the choice of treatment should balance mycotoxin reduction with preservation of nutritional and functional properties, such as solubility, emulsifying capacity and foaming ability. Moreover, selecting clays with high affinity for polar mycotoxin groups and optimizing enzymatic hydrolysis conditions can enhance detoxification efficiency. Overall, these approaches offer practical, scalable solutions for producing safer protein concentrates from contaminated raw materials.

However, the studies included in this systematic review, namely Oakes et al. [55], White et al. [56] and Tan et al. [61], did not specify whether the initial contamination in the raw materials was natural or intentional. This is a critical factor, as the nature of the contamination may influence the binding mechanisms, detoxification efficiency and interactions with the protein matrix. Addressing this gap in future research is essential to validate the effectiveness of mitigation strategies under realistic contamination conditions and to ensure that protein products are both safe and functionally suitable for food applications.

## 4. Conclusions

Available data indicates that, despite the increase in techniques and sources for producing protein concentrates and isolates, there is insufficient information on mycotoxin profiles. Methodological limitations in the current literature include the predominance of small-scale or experimental studies, variability in analytical methods for mycotoxin detection and limited reporting on the effects of specific pre-treatments or processing parameters.

Research gaps identified include the limited exploration of chemical and enzymatic treatments for mycotoxin decontamination, as well as insufficient studies connecting food safety, product quality, and potential applications of plant-based protein concentrates. While aflatoxins have been the main focus due to their toxicity and prevalence, other mycotoxins remain under-investigated.

Recommendations for future work include the following: (1) evaluating how different protein extraction and pretreatment methods influence mycotoxin levels and bioaccessibility; (2) developing standardized methodologies for mycotoxin analysis in protein concentrates; and (3) conducting studies that integrate safety, quality, and functional properties to support the development of new safe plant-based products. These efforts are essential to mitigate mycotoxin exposure and ensure the safety of plant-based protein ingredients for human consumption.

## 5. Material and Methods

### 5.1. Search Strategy Protocol

This systematic review was conducted retrospectively and was not registered prospectively in PROSPERO, Open Science Framework or INPLASY prior to the literature search. However, the methodology followed the PRISMA 2020 guidelines to ensure transparency and rigor. The strategy for structuring a systematic review uses the PICO model, which helps develop a review question and ensures that the relevant components are well defined. PICO is an acronym for Population, Intervention, Comparison and Outcome. It is designed in the research protocol phase and determines the eligibility criteria for inclusion and exclusion from the review [60,68]. In this systematic review, the PICO components were defined as follows: (1) population: mycotoxins; (2) intervention: protein concentrates; (3) comparison: protein isolates; (4) outcome: occurrence of mycotoxins in protein concentrates and isolates.

This study investigated mycotoxin contamination in protein concentrates and isolates from plant sources and reduction in their levels by applied pre-treatments, guided by the following question: “Is there an occurrence of mycotoxins in protein concentrates and isolates?”. The databases used for the search were Scopus, Web of Science and Science Direct, from 2010 to 2024 (14 years). The bibliographic research was conducted on 29 April 2024.

The following words were searched: protein isolate AND mycotoxin, protein concentrate AND protein isolate AND mycotoxin, protein concentrate AND protein isolate AND mycotoxin, protein concentrate AND mycotoxin AND occurrence, protein isolate AND mycotoxin AND occurrence AND protein concentrate AND protein isolate AND mycotoxin AND occurrence. Additionally, advanced search functions were employed to refine the query results.

### 5.2. Bibliometric Analysis

Bibliometric analysis was conducted using the Rayyan Intelligent Systematic Review app [69]. Papers found in the databases were independently evaluated by two researchers using Rayyan. Disagreements were resolved through discussion and consensus; when consensus could not be reached, a third researcher made the final decision.

Using VOS viewer software version 1.6.20, word clouds were generated from the library of papers exported from Rayyan based on titles in the screening stage. The minimum number of keyword occurrences was set to five (Figure 2).

### 5.3. Inclusion and Exclusion Criteria

Studies included in this systematic review met the following inclusion criteria: (a) focus on the production of protein concentrates, (b) focus on the production of protein isolates and (c) focus on the occurrence of mycotoxins in protein concentrates and isolates. Studies on mycotoxins in other types of food published in books, workshops, clinical trials, theses, dissertations, review articles, and case reports were excluded.

### 5.4. Data Extraction

Data extracted from the papers included first author’s name, year of publication, type of mycotoxin, type of protein source, mean or median concentrations, and reduction in mycotoxin levels.

## Figures and Tables

**Table 1:** Data extracted from papers selected in the systematic review.

Mycotoxin	Sample	Pretreatment	Concentration	LOQ	Reduction	Reference
AF	Peanut meal	-	59 μg kg^−1^	1 μg kg^−1^	-	[55]
AF	Peanut meal protein hydrolysate	pH 2.1	34.8 μg kg^−1^		41.0%	
AF	Peanut meal protein hydrolysate	pH 2.1 and 2% AB20A	7.2 μg kg^−1^		87.8%	
AF	Peanut meal protein hydrolysate	1% Pepsin	16.1 μg kg^−1^		72.7%	
AF	Peanut meal protein hydrolysate	1% Pepsin and 2% AB20A	5.1 μg kg^−1^		91.3%	
AF	Peanut meal protein hydrolysate	pH 9.1	20.1 μg kg^−1^		65.9%	
AF	Peanut meal protein hydrolysate	pH 9.1 and 2% AB20A	ND		-	
AF	Peanut meal protein hydrolysate	11.5% Alcalase	10.8 μg kg^−1^		81.7%	
AF	Peanut meal protein hydrolysate	11.5% Alcalase and 2% AB20A	0.2 μg kg^−1^		99.0%	
AF	Peanut meal (PM)	-	191.0 μg kg^−1^	1 μg kg^−1^	-	[56]
AF	Peanut meal protein hydrolysates	liquid hydrolysate + 4% Novasil + centrifugation (4500× *g* for 15 min) + 4% Novasil	2.3 μg kg^−1^		98.8%	
AF	Peanut meal protein hydrolysates	liquid hydrolysate + 4% Novasil + centrifugation (4500× *g* for 15 min) + 8% Novasil	2.7 μg kg^−1^		98.5%	
AF	Peanut meal protein hydrolysates	liquid hydrolysate + 4% AB20 + centrifugation (4500× *g* for 15 min) + 4% AB20	13.6 μg kg^−1^		92.8%	
AF	Peanut meal protein hydrolysates	liquid hydrolysate + 4% AB20+ centrifugation (4500× *g* for 15 min) + 8% AB20	11.4 μg kg^−1^		94.0%	
ZEN	Corn gluten meal (CGM)	-	5500 μg kg^−1^ *		-	[61]
ZEN	Zein	pH 14 to 40 °C	4800 μg kg^−1^ *	8.5 μg kg^−1^	12.7%	
ZEN	Zein	pH 14 to 80 °C	2000 μg kg^−1^ *		63.6%	

LOQ = Limit of quantification; AB20A = Bentonite clay; * = approximate values; - = not estimated. ND: Not detected.

## Data Availability

No new data were created or analyzed in this study. Data sharing is not applicable to this article.

## Abbreviations

AFS	Aflatoxins
FBs	Fumonisins
ZEN	Zearalenone
TCT	Trichothecenes
PAT	Patulin
DON	Deoxynivalenol
PF	Peanut Flour
